# Compound phellodendron decoction promotes post-fistulotomy wound healing by downregulating neutrophil extracellular traps formation: evidence from a randomized trial and mechanistic studies

**DOI:** 10.3389/fphar.2026.1732119

**Published:** 2026-09-15

**Authors:** Ju Wang, Heng Deng, Shuo Chen, Hui Liu

**Affiliations:** 1 Anorectal Surgery, Lu’an City Hospital of Chinese Medicine, Lu’an, China; 2 Department of Anorectal Surgery, Second Affiliated Hospital, Anhui University of Chinese Medicine, Hefei, China; 3 Department of Anorectal Surgery, Beijing University of Chinese Medicine Shenzhen Hospital (Longgang), Shenzhen, China; 4 General Practice Department, Anhui Provincial Second People’s Hospital, Hefei, China

**Keywords:** compound phellodendron decoction, neutrophil extracellular traps, pad4, randomized controlled trial, wound healing

## Abstract

**Background:**

Persistent inflammation and dysregulated neutrophil extracellular traps (NETs) are key factors impairing postoperative wound healing. While the traditional formula Compound Phellodendron Decoction (CPD) is clinically effective in sitz-bath therapy, its mechanism of action, particularly concerning the novel therapeutic target NETosis, remains unknown. We hypothesized that CPD accelerates healing by downregulating NET formation.

**Methods:**

In a randomized, double-blind, placebo-controlled trial, 60 patients post-anal fistulotomy received daily topical ultrasonically nebulized CPD (n = 30) or placebo (n = 30). Primary endpoints were healing time and scores for edema, pain, and exudation on days 3 and 7. Secondary outcomes included inflammatory cytokine levels, hydroxyproline, angiogenesis, and direct histological and ultrastructural assessment of NETs in granulation tissue. To dissect the underlying mechanism, we investigated the effect of CPD on NETosis in primary human neutrophils, measuring NET formation, reactive oxygen species (ROS) generation, and expression of key mediators (CitH3, PAD4).

**Results:**

CPD significantly shortened wound healing time (40.23 ± 9.61 vs. 45.84 ± 13.29 days, P < 0.001) and improved clinical scores. It modulated inflammation by elevating IL-2 and IL-10 while reducing IL-6, IL-22, and TNF-α. Critically, immunofluorescence and transmission electron microscopy revealed a substantial reduction in NETosis in CPD-treated tissues, accompanied by enhanced angiogenesis. *In vitro*, CPD dose-dependently inhibited PMA-induced NETosis, an effect mediated by reduced ROS generation and decreased PAD4 protein abundance.

**Conclusion:**

CPD facilitates wound healing through a dual mechanism involving systemic immunomodulation and attenuation of NETosis by the ROS/PAD4 axis in neutrophils. Our study identifies CPD as a promising NETosis-targeted therapeutic agent for chronic wound management.

## 1 Introduction

Postoperative wound healing following anal fistula surgery is frequently compromised by persistent inflammation and a high risk of infection (Li et al., 2022).

The anatomical peculiarity of the anal region, coupled with the open nature of the wounds (Khoshnevis et al., 2022), increases susceptibility to contamination, thereby exacerbating edema and secretion (Quinn et al., 2025), which impedes healing and increases the burden on patients (Gao et al., 2022). Sustained inflammation responses are the critical driver of delayed wound closure in this patient population (Porwal et al., 2021). Recent studies have highlighted the critical pathological role of NETosis in both acute surgical wounds and in chronic refractory tissue injury (James et al., 2024). Excessive NET formation is established as a core driver of impaired cutaneous and anorectal wound repair (Asiri et al., 2024; Zhang et al., 2025). Targeted NET inhibition represents a promising therapeutic direction for stalled surgical wounds.

Clinically, topical sitz bath with Compound Phellodendron Decoction (CPD) has long been used to relieve pain and accelerate fistulotomy wound recovery (Deng et al., 2022; Yijuan et al., 2024), yet its underlying molecular mechanism remains largely uncharacterized. Existing modern pharmacological research has preliminarily confirmed that signature metabolites extracted from each botanical drug within CPD exert regulatory effects on neutrophil activation and NET generation. Berberine, matrine and tanshinone IIA are reported to suppress ROS/PAD4-dependent NETosis in single-compound cell or animal models (Fang et al., 2025), while ginsenosides and ferulic acid alleviate NET-triggered inflammatory cytokine secretion and endothelial injury (Kang et al., 2022). Nevertheless, these preclinical findings are restricted to isolated pure metabolites, and no rigorous randomized controlled trial has explored whether the complete multi-botanical CPD formulation can regulate NETosis within human perianal postoperative wound microenvironments.

Accordingly, we designed a randomized double-blind placebo-controlled clinical trial combined with primary human neutrophil functional assays to determine whether CPD facilitates fistulotomy wound repair via suppression of the NOX2/ROS/PAD4-NETosis signaling cascade, filling the research gap between single-metabolite basic experiments and NET-targeted multi-herbal clinical intervention for anorectal surgery.

## 2 Materials and methods

This randomized, double-blind, placebo-controlled trial (ClinicalTrials ID: NCT06663527) was approved by the Ethics Committee of the Second Affiliated Hospital of Anhui University of Chinese Medicine (Approval No. 2022-zj-19-X1; Date: 29 April 2022). The study adhered to the 25-item CONSORT checklist for reporting standards (Butcher et al., 2022; Figure 1).

**FIGURE 1:**
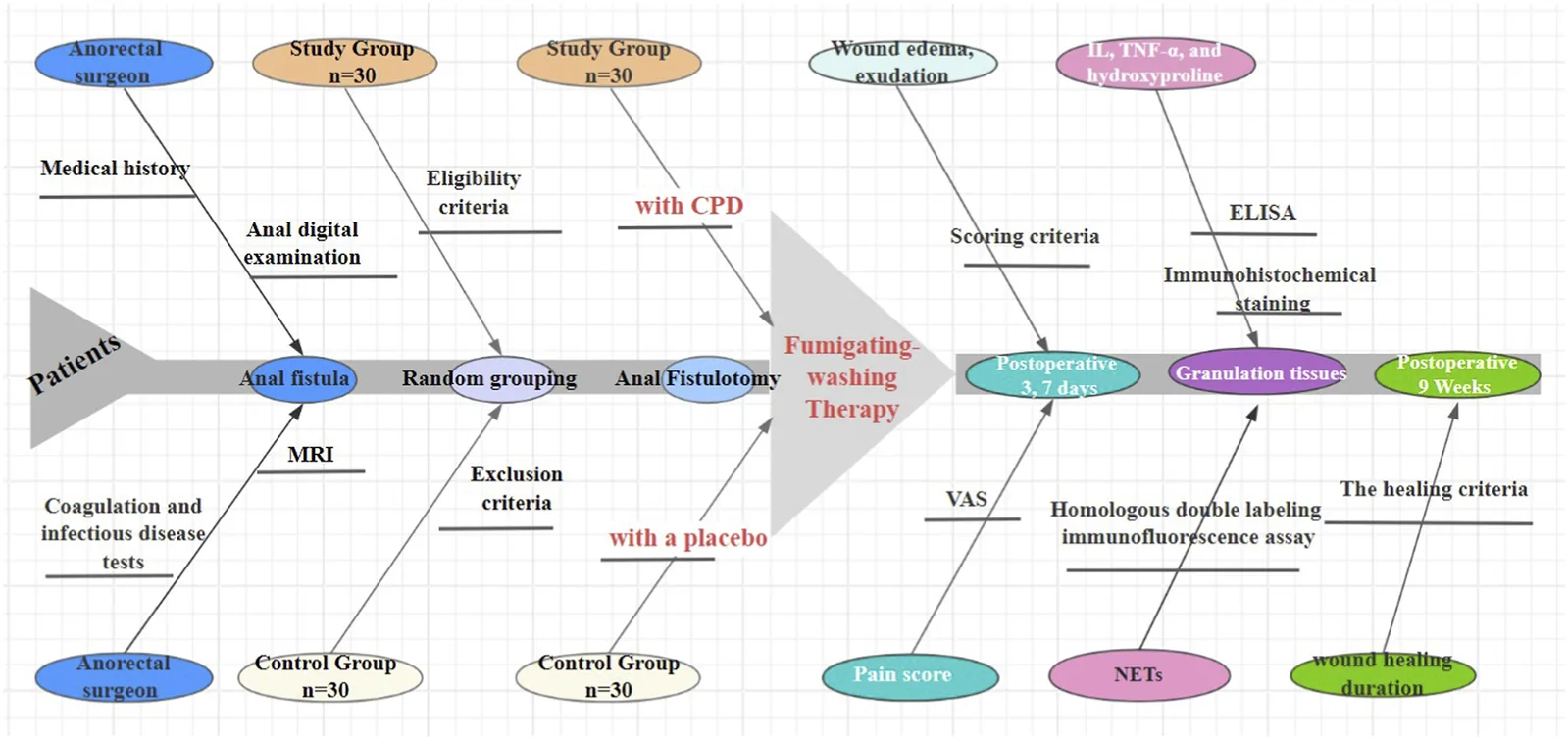
CONSORT flow diagram of the study.

### 2.1 Patients

Sample size calculation was performed prior to trial initiation based on preliminary clinical healing time data, with α = 0.05 and statistical power set to 0.80. A total of 60 participants (30 per arm) were required to detect a statistically meaningful difference in primary wound healing endpoints. Allocation was concealed using sealed envelopes. Eligibility criteria were determined for patients within the age range of 18–80 years who had undergone anal fistulotomy procedures. Exclusion criteria encompassed patients with Crohn’s disease, pregnancy or lactation, organ dysfunction (such as heart failure, respiratory insufficiency, liver or renal impairment, gastrointestinal dysfunction), coagulopathy, autoimmune diseases, or a history of hypersensitivity to Chinese medicinal substances.

### 2.2 CPD and placebo preparation

A total of 100 g of dried medicinal substances (including 30 g of *Phellodendron chinense* schneid. [Rutaceae*; phellodendri cortex*], 25 g of *Sophora flavescens Ait.* [Fabaceae*; S. flavescens Ait. radix et rhizoma*], 15 g of *Salvia miltiorrhiza Bge.* [Lamiaceae*; Salviae miltiorrhizae Bge.radix et rhizoma*], 10 g of *Panax notoginseng (Burk.) F.H.Chen* [Araliaceae*; P. notoginseng (Burk.)F.H.Chen radix et rhizoma*], and 20 g of *Angelica sinensis (Oliv.) Diels* [Apiaceae*; A. sinensis (Oliv.) Diels radix*], registered in the Chinese Pharmacopoeia) were decocted in 1 L of water to yield 400 mL of decoction using an automatic decocting machine (JMC-25L, Jimi). CPD was prepared to a concentration of 0.25 g crude botanical drug per milliliter aqueous decoction (w/v, raw drug weight basis) and stored at 4 °C.

To produce a placebo similar in appearance, scent, and color to CPD, a 200 mL placebo was formulated with the following compounds: caramel food colorant (50 g, product number: CC-03, Xinxiang, Henan), sucrose octaacetate (30 g, brand: Gaotai, product number: 2561498), maltodextrin (20 g, brand: Baolingbao, article number: ZCC2308141449), and sodium chloride injection (190 mL). Placebo metabolites were confirmed inert in pilot tests. All herbal materials were authenticated by Pengcheng Liu from Lu’an Hospital of Chinese Medicine. All solid excipients were fully dissolved under constant stirring and the final volume was calibrated to 200 mL with normal saline to eliminate volume discrepancy; pilot cell assays confirmed this placebo formulation exerts no anti-inflammatory or pro-healing bioactivity.

### 2.3 Quality control and standardization of CPD by UPLC-MS

Microbiological quality control of both CPD and placebo preparations was performed in accordance with the Chinese Pharmacopoeia (2020 Edition). All batches met the accepted sterility criteria for topical applications (aerobic microbial count <100 CFU/mL; yeast and mold <10 CFU/mL).

To ensure batch-to-batch consistency and validate the presence of key bioactive compounds, the chemical profile of CPD was chemically fingerprinted using ultra-performance liquid chromatography-mass spectrometry (UPLC-MS). The analysis confirmed the presence and quantity of six metabolites with established efficacy in wound healing: berberine (4.4 ± 0.61 mg/g), matrine (21.3 ± 2.43 mg/g), tanshinone IIA (19.9 ± 4.87 mg/g), ginsenoside Rg1 (79.6 ± 7.43 mg/g), ferulic acid (0.67 ± 0.071 mg/g), and phenylethanol glycoside (7.3 ± 0.64 mg/g). This quality control step ensured that all batches of CPD used in the trial were chemically identical and of high potency. The detailed UPLC-MS chromatograms, mass spectra, and validation data for the quantification of bioactive compounds (berberine, matrine, tanshinone IIA, ginsenoside Rg1, ferulic acid, and phenylethanol glycoside) are provided as Supplementary File S1.

### 2.4 Topical vapor therapy

All patients underwent anal fistulotomy based on the Parks classification (Emile et al., 2021). Commencing the day following surgery, topical vapor therapy was conducted once daily, either post-defecation or 2 hours before intravenous infusion. In the CPD group, 200 mL of CPD was mixed with 2 L of water at 40 °C and used in an ultrasonic atomizing device (Model: TM50-C, Xuzhou Tianma Medical Equipment Factory, China) that was classified by the FDA as Class II (special controls) (Food and Drug Administration, 2005). Ultrasonic atomization was performed at 1.7 MHz frequency and 40 mL/min flow rate (device specifications from the manufacturer’s manual). Patients sat 15 cm from the nozzle, fully exposing the perianal wound to the nebulized solution for 12 min per session. Treatment adherence was monitored by recording the returned volume of solution and patient diaries (Supplementary Video S1).

### 2.5 Therapeutic effect and primary endpoints

Wound edema, exudation, and local pain were evaluated on postoperative days 3 and 7. Two independent surgeons evaluated edema and exudation using a standardized protocol. Inter-rater reliability was confirmed by Cohen’s kappa (>0.8) during pre-trial training. Scores were defined as: 0 (none), 1 (<25% coverage), 2 (25%–50%), 3 (>50%). Edema and exudation were scored as: 0 (none), 1 (<1/4 wound coverage), 2 (1/4–1/2 coverage), or 3 (>1/2 coverage) (Deng et al., 2022). Pain intensity was assessed using a Visual Analogue Scale (VAS; 0–10, 0 = no pain). Time to complete wound healing was recorded, defined by: (1) full scar tissue coverage, (2) absence of secretion or exudate, and (3) no detectable difference between scar and adjacent tissue, with no pain on digital examination.

### 2.6 Enzyme-linked immunosorbent assay (ELISA)

Levels of IL-2, IL-6, IL-10, IL-22, TNF-α, and hydroxyproline in granulation tissue homogenates were quantified using commercial ELISA kits (Jilide Biotechnology) according to the manufacturer’s instructions.

### 2.7 Immunofluorescence

As previously described (Deng et al., 2025), fresh granulation tissue sections were fixed, permeabilized, and incubated with primary antibodies against CD66b (1:200, Abcam, ab5405) and CitH3 (1:500, Abcam, ab5103) overnight at 4 °C, followed by appropriate secondary antibodies. Nuclei were counterstained with DAPI. NETs formation was detected and imaged under a fluorescence microscope (Nikon 80i). Three random high-magnification fields (400x) per slice were analyzed. All quantitative signals from multiple random fields obtained from a single patient were averaged to generate one independent data point to avoid pseudoreplication, and all image quantification was performed under blinded evaluation.

### 2.8 Transmission electron microscopy (TEM)

Granulation tissue samples (n = 5 per group) were fixed in glutaraldehyde, post-fixed in osmium tetroxide, and processed for standard transmission electron microscopy analysis.

### 2.9 Hematoxylin and eosin (HE) staining

For histological analysis, sections were stained with Hematoxylin and Eosin (HE) or processed for immunohistochemistry using an anti-CD31 antibody (1:100, Shanghai Rongxin Biotechnology, 20210068) following standard protocols.

### 2.10 Immunohistochemical staining

Immunohistochemistry was performed on granulation tissue sections using a standard protocol. After antigen retrieval, sections were incubated with a primary antibody against CD31 (1:100, Shanghai Rongxin Biotechnology, 20210068) overnight at 4 °C. This was followed by incubation with an HRP-conjugated secondary antibody and development with 3,3′-diaminobenzidine (DAB) chromogenic solution. Sections were counterstained with hematoxylin. Images of three random high-magnification fields (400x) per slice were captured using a Nikon 80i microscope for subsequent quantification.

### 2.11 *In vitro* studies on human neutrophils

#### 2.11.1 Cell culture and preparation of CPD microfiltrate

Human peripheral blood neutrophils (STEMCELL Technologies, #70025) were cultured in complete RPMI-1640 medium. Cell viability exceeded 95% after thawing. The stock CPD decoction was centrifuged and sterile-filtered (0.22 μm) to obtain CPD microfiltrate for cell treatments.

#### 2.11.2 Determination of non-cytotoxic concentrations

Neutrophils were treated with CPD microfiltrate (0–400 μg/mL) for 6 h. Cell viability was assessed using a CCK-8 kit. Concentrations of 50, 100, and 200 μg/mL, which showed no cytotoxicity, were selected for subsequent experiments.

#### 2.11.3 Experimental groups and NETosis induction

Neutrophils were seeded into culture plates and allowed to adhere for 1 h at 37 °C with 5% CO_2_. Cells were then assigned to the following groups and pre-treated for 2 h:

Control group: Culture medium only.
PMA group: Culture medium only (to be induced with PMA).

CPD-L, CPD-M, CPD-H groups: CPD microfiltrate at the non-cytotoxic concentrations of 50, 100, and 200 μg/mL, respectively.

Si-NOX2 group: Cells were transfected with NOX2-specific siRNA using the Lipofectamine™ RNAiMAX transfection reagent according to the manufacturer’s instructions.

Following pre-treatment, NETosis was induced in all groups except the Control group by adding phorbol 12-myristate 13-acetate (PMA, Sigma-Aldrich, United States) to a final concentration of 100 nM (Atteberry et al., 2024). The cells were then incubated for an additional 4 h before subsequent analysis.

#### 2.11.4 Assessment of NETosis

##### 2.11.4.1 Immunofluorescence staining

After treatments, cells were fixed with 4% paraformaldehyde, permeabilized, and subjected to immunofluorescence staining. NETs were labeled using a rabbit anti-human Citrullinated Histone H3 (CitH3) antibody (Abcam, ab5103) and a mouse anti-human neutrophil elastase (NE) antibody (Santa Cruz Biotechnology, sc-55549), followed by Alexa Fluor 488- and 594-conjugated secondary antibodies, respectively. Nuclei were counterstained with DAPI. Images were captured using a Nikon A1R confocal microscope, and PMA stimulation induced massive extracellular DNA extrusion from isolated human neutrophils, clearly traced via dual CitH3 and NE fluorescent labeling.

##### 2.11.4.2 Quantification of extracellular DNA

NETosis was quantitatively assessed by measuring extracellular DNA release using the Quant-iT PicoGreen dsDNA Assay Kit (Invitrogen), following the manufacturer’s protocol. Fluorescence was measured with a microplate reader (BioTek) at 480/520 nm excitation/emission (Li et al., 2025).

#### 2.11.5 Measurement of intracellular reactive oxygen species (ROS)

Intracellular ROS levels were detected using the fluorescent probe DCFH-DA (Beyotime Biotechnology). After treatments, cells were incubated with 10 μM DCFH-DA at 37 °C for 30 min. Fluorescence intensity was analyzed using a BD Accuri C6 flow cytometer (BD Biosciences), with a minimum of 10,000 events collected per sample. Data were analyzed using FlowJo software (v10.8, BD Biosciences).

#### 2.11.6 Western blot analysis

After treatments, cells were lysed and proteins were extracted. Proteins were separated by SDS-PAGE, transferred to PVDF membranes, and probed with primary antibodies against NOX2 (abcam, ab129068), phospho-NF-κB p65 (Cell Signaling Technology, #3033), total NF-κB p65 (Cell Signaling Technology, #8242), PAD4 (Abcam, ab214810), and β-Actin (Cell Signaling Technology, #4970S). After incubation with HRP-conjugated secondary antibodies, bands were visualized using an ECL substrate and quantified with ImageJ software (National Institutes of Health).

### 2.12 Statistical analysis

Data are presented as mean ± SD. Group comparisons were performed using independent t-tests, Mann-Whitney U, χ^2^, or repeated-measures ANOVA with Bonferroni correction, as appropriate. A p-value < 0.05 was considered significant. For multiple pairwise comparisons between time points and experimental groups, the Bonferroni correction was applied to control type I error inflation. Mean difference and 95% confidence intervals (95% CI) between CPD and placebo groups were calculated and presented in Supplementary Table S2.

## 3 Results

### 3.1 Patient characteristics and clinical efficacy of CPD

All 60 enrolled patients completed the study and were included in the analysis (no dropouts). No treatment-related adverse events were observed. Sixty patients (51 males, 9 females; mean age 40.23 ± 12.61 years, range 18–80) were included, with 18 high anal fistula and 42 low anal fistula cases. The control group comprised 26 males and 4 females (mean age 39.23 ± 11.96 years, range 18–78), while the CPD group included 25 males and 5 females (mean age 41.24 ± 10.51 years, range 22–80) (Table 1). No significant differences were found between groups in gender (χ^2^ = 2.141, P = 0.265), age (T = 0.812, P = 0.398), or other baseline characteristics. No treatment-related adverse events (e.g., allergic reactions or wound irritation) were reported in either group, consistent with the established safety profile of topical herbal therapies. This supports the clinical feasibility of CPD atomization therapy.

**TABLE 1:** General clinical data.

Item	CPD group (*n* = 30)	Control Group (*n* = 30)
Gender (male/female)	25/5	26/4
Age	41.24 ± 10.51	39.23 ± 11.96
Length of fistula (cm)	3.2 ± 0.5	3.1 ± 0.6
Fistula type (low/high)	21/9	21/9
Operation time (min)	29.5 ± 5.3	28.2 ± 4.8

On postoperative days 3 and 7, the CPD group demonstrated significantly lower wound edema, pain, and exudation scores compared to the control group (P < 0.001) (Table 2). Although all patients achieved complete wound healing, the healing process was significantly accelerated in the CPD group (40.23 ± 9.61 days) compared to the control group (45.84 ± 13.29 days) (P < 0.001).

**TABLE 2:** Clinical outcomes and inflammatory biomarker levels in the study and control groups.

Outcome measures	Group	Post-op day 3	Post-op day 7	Main effect: group (G) F (P)	Main effect: time (T) F (P)	Interaction: G × T F (P)	Post-hoc analysis (bonferroni)
Edema score	CPD	2.02 ± 0.33	1.56 ± 0.27	F (1,58) = 15.2 (<0.001)	F (1,58) = 125.4 (<0.001)	F (1,58) = 4.15 (0.046)	^†‡^
	Control	2.30 ± 0.21	1.86 ± 0.29				
Pain score (VAS)	CPD	6.36 ± 1.04	3.98 ± 0.87	F (1,58) = 12.8 (<0.001)	F (1,58) = 185.3 (<0.001)	F (1,58) = 2.59 (0.113)	^†^
	Control	7.82 ± 1.73	5.09 ± 1.07				
Exudation score	CPD	2.26 ± 0.41	1.35 ± 0.29	F (1,58) = 18.5 (<0.001)	F (1,58) = 210.8 (<0.001)	F (1,58) = 9.48 (0.003)	^†‡^
	Control	2.82 ± 0.47	1.77 ± 0.36				
IL-2 (ng·g^−1^)	CPD	46.17 ± 5.79	86.65 ± 8.15	F (1,58) = 90.5 (<0.001)	F (1,58) = 895.2 (<0.001)	F (1,58) = 25.3 (<0.001)	^†‡^
	Control	35.49 ± 5.37	64.19 ± 8.36				
IL-6 (ng·g^−1^)	CPD	48.17 ± 5.86	34.12 ± 10.24	F (1,58) = 45.8 (<0.001)	F (1,58) = 98.3 (<0.001)	F (1,58) = 18.8 (<0.001)	^†‡^
	Control	69.99 ± 9.49	46.78 ± 5.71				
IL-10 (ng·g^−1^)	CPD	5.22 ± 0.60	12.95 ± 2.42	F (1,58) = 95.1 (<0.001)	F (1,58) = 210.5 (<0.001)	F (1,58) = 105.6 (<0.001)	^†‡^
	Control	4.73 ± 0.97	6.04 ± 0.83				
TNF-α (ng·g^−1^)	CPD	87.41 ± 4.36	60.06 ± 11.86	F (1,58) = 55.8 (<0.001)	F (1,58) = 180.2 (<0.001)	F (1,58) = 8.56 (0.005)	^†‡^
	Control	100.89 ± 5.95	77.25 ± 12.92				​
Hydroxyproline (μg·mg^−1^)	CPD	0.955 ± 0.166	1.90 ± 0.209	F (1,58) = 102.3 (<0.001)	F (1,58) = 950.1 (<0.001)	F (1,58) = 89.4 (<0.001)	^†‡^
	Control	0.846 ± 0.153	1.103 ± 0.145				​
Healing time (days)	CPD	40.23 ± 9.61		t (58) = 3.45, P = 0.001	—	—	—
	Control	45.84 ± 13.29					

Data are presented as mean ± SD., Data were analyzed by two-way repeated-measures ANOVA (RM-ANOVA). The test statistic reported is the F-value with degrees of freedom (df) for the Group × Time interaction effect (df = 1, 58 for all measures). Healing time was compared using an independent samples *t*-test. Post-hoc Analysis Symbols: ^†^The CPD, group is significantly different from the Control group at the same time point (P < 0.05); ^‡^The value at Day 7 is significantly different from Day 3 within the same group (P < 0.05). Mean differences and 95% CI, for primary healing endpoint are provided in Supplementary Table S2.

### 3.2 CPD modulates the inflammatory microenvironment and suppresses NETosis in wound tissue

#### 3.2.1 CPD ameliorates the inflammatory cytokine milieu

Levels of IL-2, IL-10, and hydroxyproline in granulation tissue increased by postoperative day 7 compared to day 3 in both groups, while IL-6, IL-22, and TNF-α levels significantly decreased. The CPD group showed higher IL-2, IL-10, and hydroxyproline levels and lower IL-6, IL-22, and TNF-α levels compared to the control group on both days 3 and 7 (P < 0.001) (Table 2; Figure 2).

**FIGURE 2:**
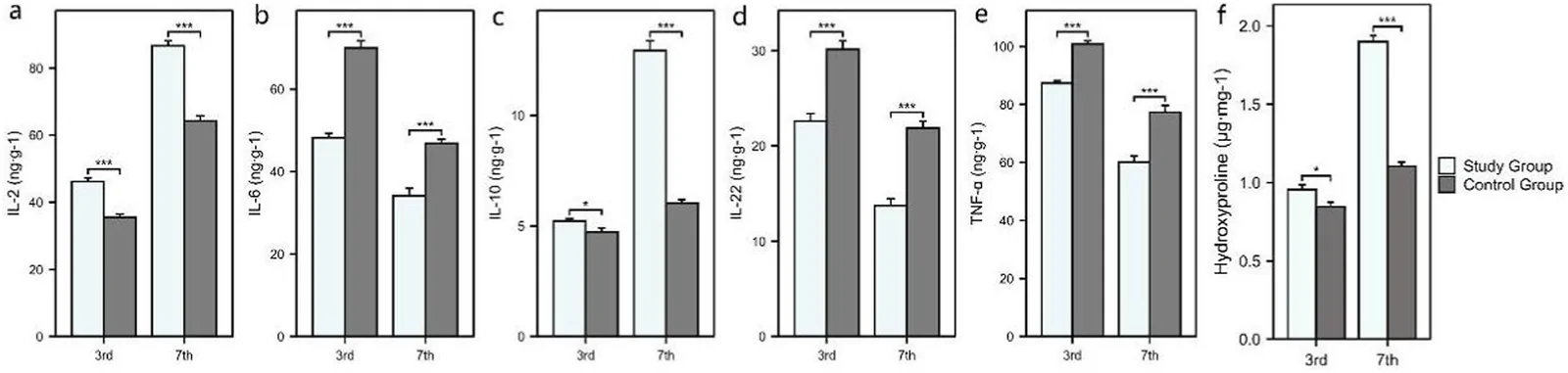
CPD modulates the inflammatory microenvironment in wound granulation tissue. Levels of **(a)** IL-2, **(b)** IL-6, **(c)** IL-10, **(d)** IL-22, **(e)** TNF-α, and **(f)** hydroxyproline were measured by ELISA in granulation tissues from the CPD and control groups at postoperative days 3 and 7. Data are presented as mean ± SD (n = 30 patients per group). Statistical significance was determined by two-way repeated-measures ANOVA with Bonferroni’s *post hoc* test. *P < 0.05, ***P < 0.001.

#### 3.2.2 CPD reduces NETosis in human granulation tissue

CitH3/CD66b dual-positive mesh-like extracellular scaffolds accumulated abundantly within control granulation tissue, representing tissue-resident NET deposits, which were markedly reduced in CPD-treated tissues (Figure 3A). Quantitative analyses corroborated these findings, demonstrating that CPD treatment significantly suppressed both the abundance of CitH3+ NETs and the CitH3+/DAPI + area ratio in granulation tissue compared to the control group at postoperative days 3 and 7 (all P < 0.001; Figure 3B). While NETosis signals naturally declined from day 3 to day 7 in both groups, this resolution was significantly accelerated in the CPD-treated wounds.

**FIGURE 3:**
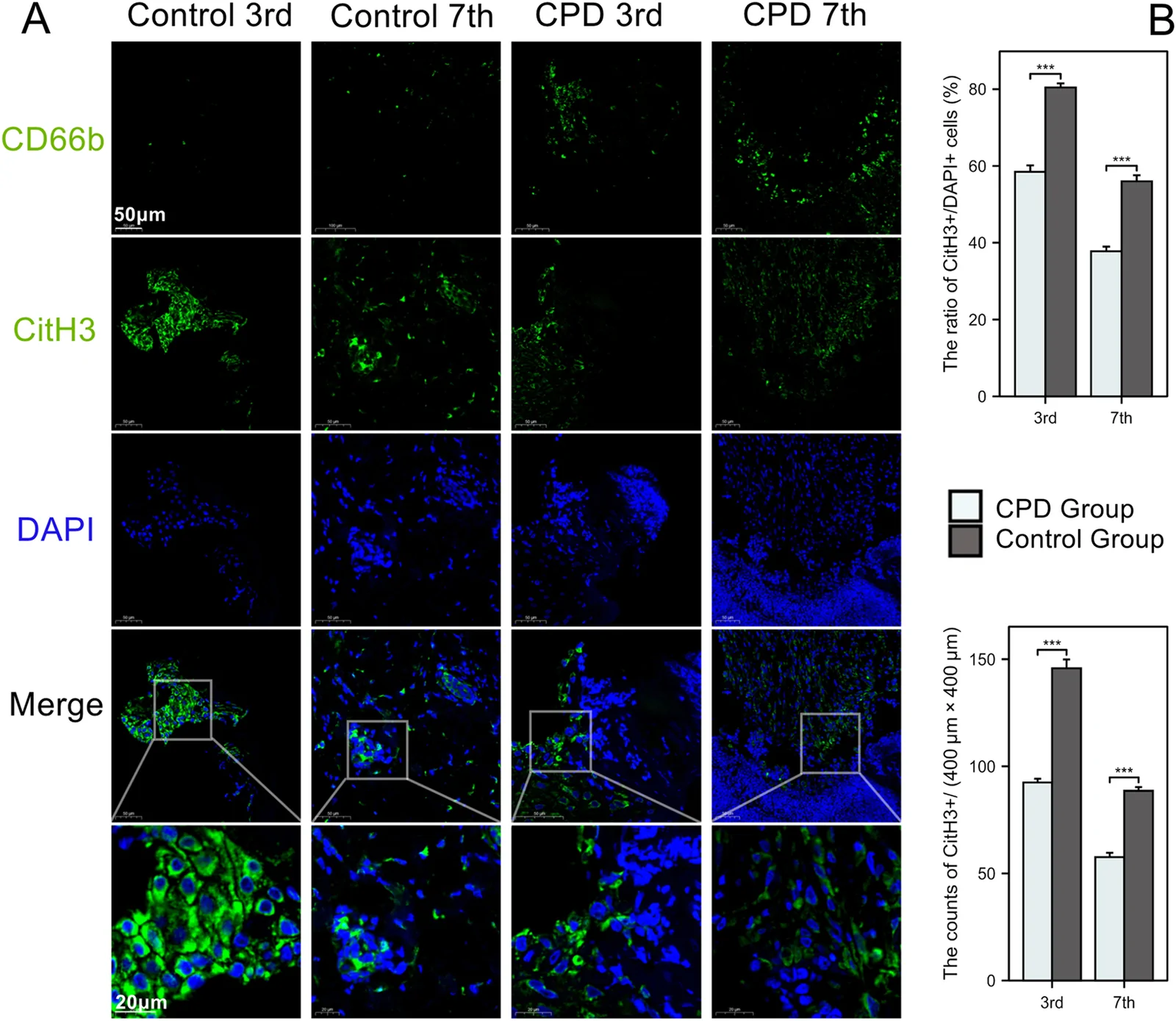
CPD inhibits NETosis in human granulation tissue. **(A)** Representative immunofluorescence images of granulation tissue sections from the CPD and control (Ctrl) groups stained for the neutrophil marker CD66b (green), citrullinated histone H3 (CitH3, green), and DAPI (blue). White arrows point to NET-like structures (CitH3+ fibers). **(B)** Quantitative analysis of CitH3+ integrated optical density (IOD) from 90 random high-power fields per group (n = 15–20 patients). Data are mean ± SD. ***P < 0.001 versus Ctrl group at the same time point (two-way ANOVA with Bonferroni’s *post hoc* test).

#### 3.2.3 Ultrastructural evidence confirms the suppression of NETosis

Transmission electron microscopy (TEM) analysis of day 7 granulation tissue provided supporting ultrastructural evidence of NETosis suppression by CPD. Neutrophils in the control group were frequently observed undergoing NETosis, characterized by plasma membrane rupture, nuclear envelope disintegration, and the extrusion of decondensed chromatin into the extracellular space (Figures 4A,C). In contrast, neutrophils from CPD-treated wounds overwhelmingly displayed an intact cellular morphology, with preserved plasma and nuclear membranes, and were rarely associated with NET-like structures (Figures 4B,D). The scarcity of such NETotic events in the CPD group provides direct visual confirmation that CPD intervention effectively curtails NETosis at the cellular level.

**FIGURE 4:**
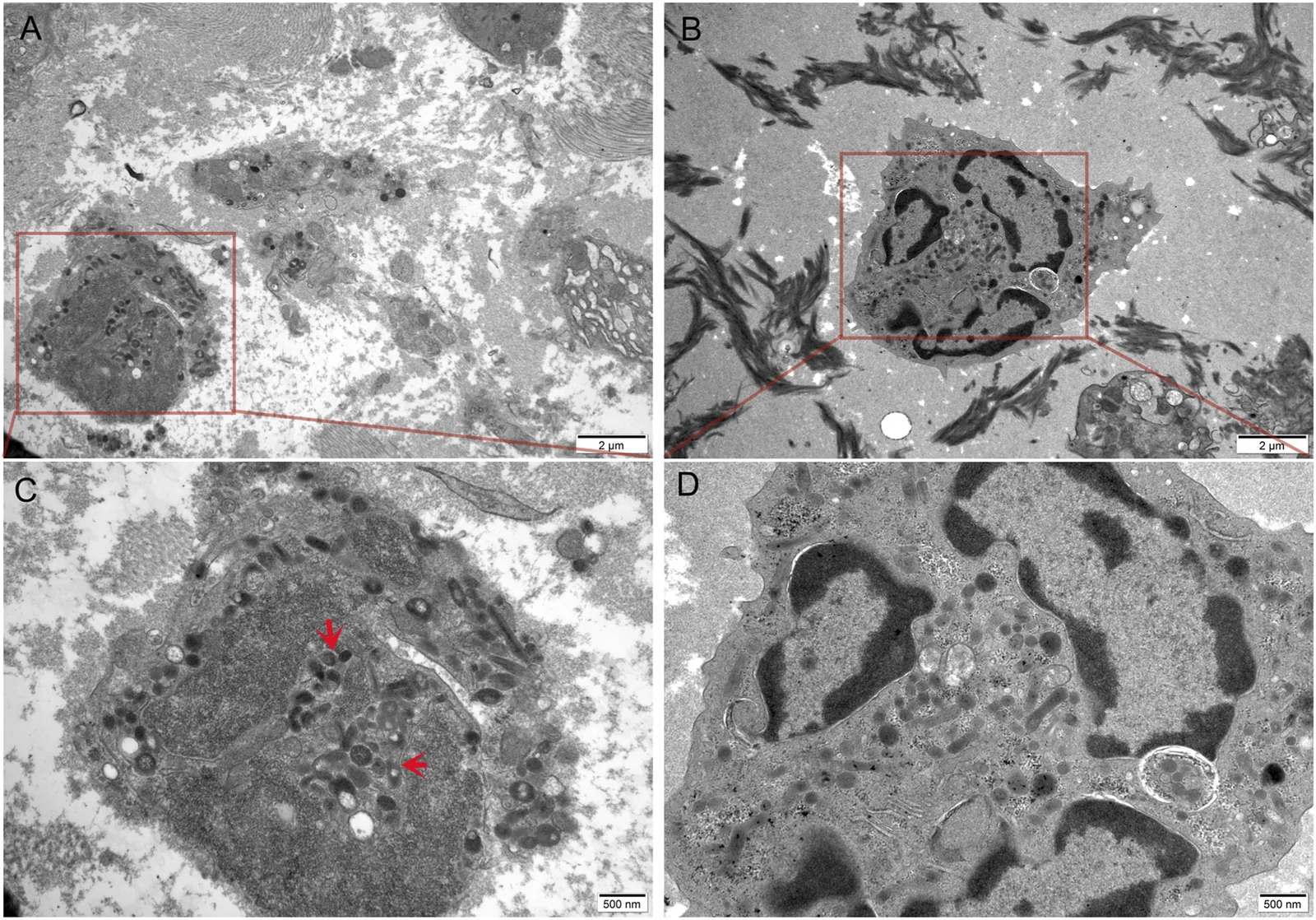
Ultrastructural evidence of CPD-mediated suppression of NETosis. **(A, C)** Representative transmission electron microscopy (TEM) images from the control group showing neutrophils undergoing NETosis, characterized by plasma membrane rupture [white arrowhead in **(A)**], nuclear decondensation, and the release of web-like chromatin structures (red arrows) decorated with globular proteins [red arrow in **(C)**]. **(B, D)** Representative TEM images from the CPD-treated group showing neutrophils with intact cellular and nuclear membranes [white arrowheads in **(B)** and **(D)**]. Scale bars: 2 μm **(A, B)**; 500 nm **(C, D)**.

### 3.3 CPD promotes angiogenesis in healing wounds

Capillary density increased in both groups by day 7 versus day 3 (P < 0.001), with higher counts in the CPD group at both timepoints versus controls (P < 0.001) (Figures 5A,B). Similarly, CD31^+^ endothelial cell counts were significantly elevated in the CPD group compared to controls on day 3 and day 7 (P < 0.001) (Figures 5C,D).

**FIGURE 5:**
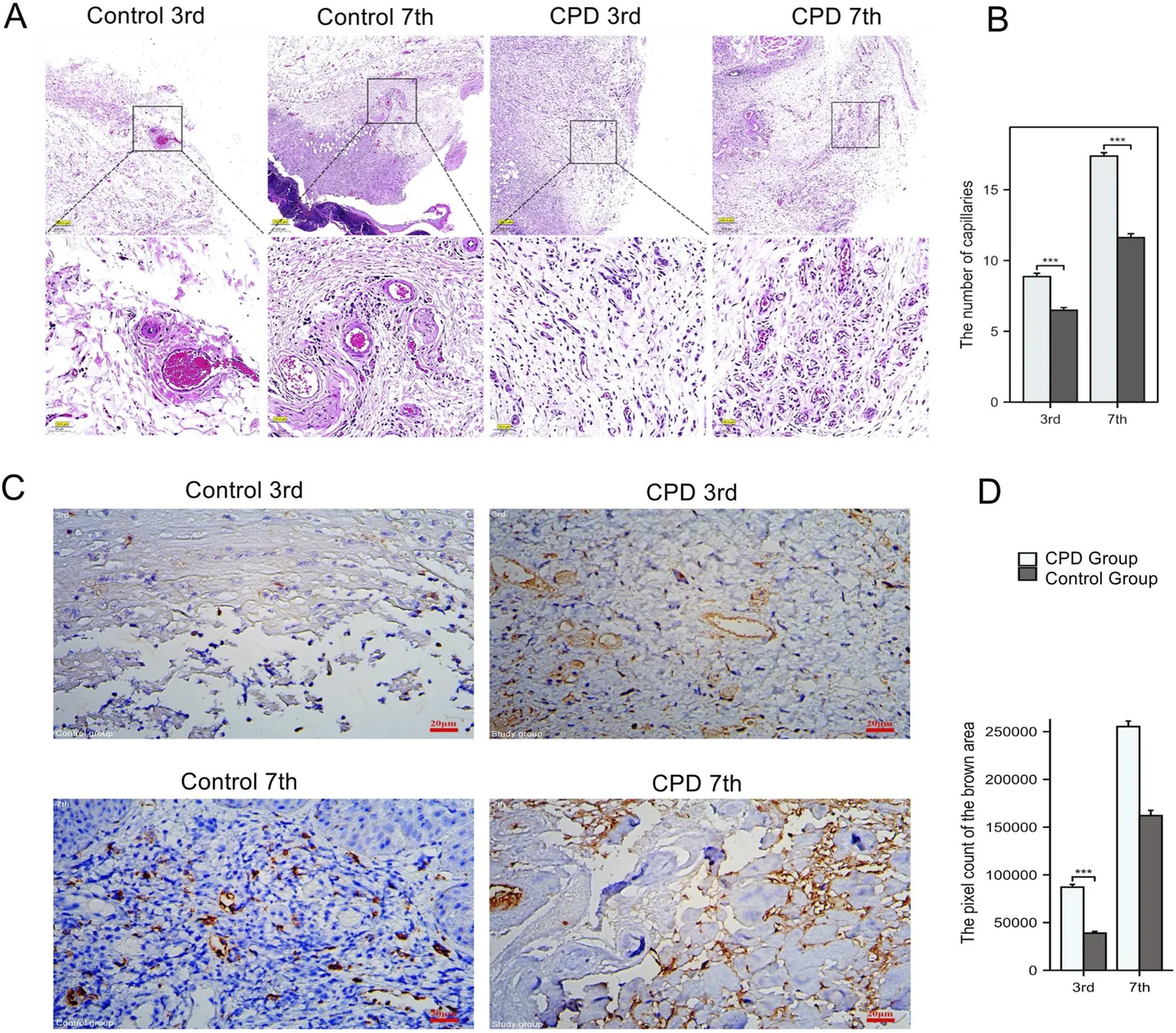
CPD promotes angiogenesis in wound granulation tissue. **(A)** Representative hematoxylin and eosin (H&E) stained sections. **(B)** Quantitative analysis of capillary density (number of capillaries per high-power field, ×400 magnification; 90 fields/group). **(C)** Representative immunohistochemical staining for CD31 (brown) highlighting vascular endothelial cells. **(D)** Quantitative analysis of CD31-positive areas presented as integrated optical density (IOD) per field (×400 magnification; 90 fields/group). Data are mean ± SD. *P < 0.001 versus control group at the same time point (two-way ANOVA with Bonferroni’s *post hoc* test for **(B)**; unpaired two-tailed Student’s t-test for **(D)**.

### 3.4 CPD downregulates NETosis via NOX2-derived ROS and the downstream PAD4 pathway

#### 3.4.1 CPD suppresses NETosis without cytotoxicity and targets NOX2

CCK-8 assay confirmed that CPD at 50, 100, and 200 μg/mL did not significantly affect neutrophil viability, whereas 400 μg/mL exhibited cytotoxicity (75.8% ± 6.7%, P < 0.01; Figure 6A). At non-toxic concentrations, CPD dose-dependently inhibited PMA-induced NETosis. Quantification of extracellular DNA release showed that PMA stimulation increased NETosis by approximately 5.1-fold over the control (P < 0.001). This was significantly suppressed by CPD treatment, with reductions of 28%, 47%, and 70% at low, medium, and high doses, respectively (all P < 0.01 vs. PMA group; Figure 6B). Genetic knockdown of NOX2 (Si-NOX2) similarly and potently inhibited NETosis, reducing extracellular DNA by 72% (P < 0.001), an inhibitory phenotype comparable to the high dose of CPD (Figure 6B).

**FIGURE 6:**
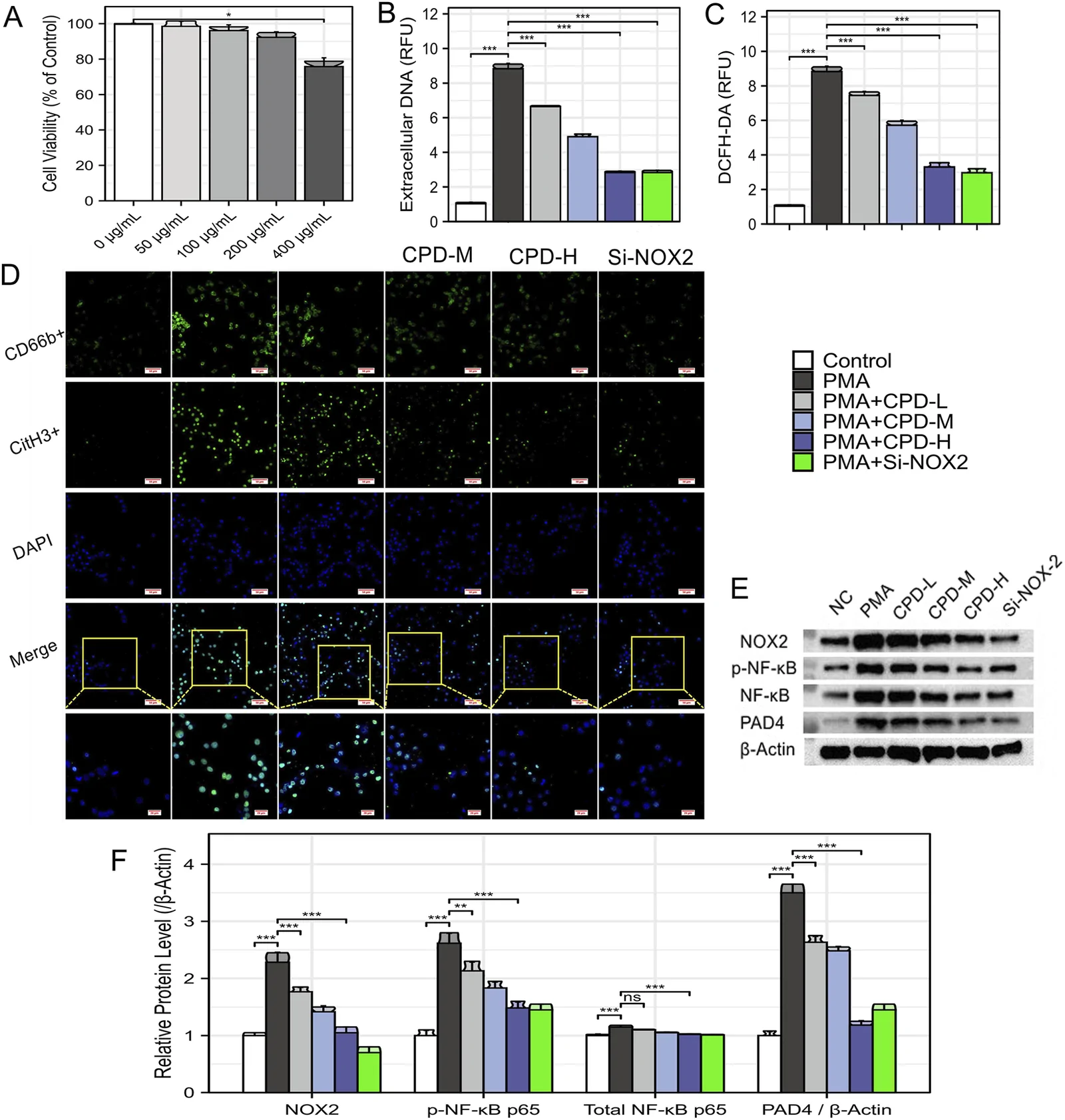
CPD downregulates PMA-induced NETosis in human neutrophils via the ROS/NF-κB/PAD4 axis. **(A)** Neutrophil viability assessed by CCK-8 assay after treatment with the indicated concentrations of CPD for 6 h. **(B)** Quantification of NETosis by measuring extracellular DNA release using PicoGreen assay. **(C)** Intracellular ROS levels measured by flow cytometry using DCFH-DA probe. **(D)** Representative immunofluorescence images of NETs stained for CitH3 (green) and DAPI (blue). **(E)** Representative Western blot analyses of phospho-NF-κB p65 (p-p65), total NF-κB p65 (t-p65), PAD4, and β-actin. **(F)** Densitometric quantification of protein levels from **(E)**. Data are presented as mean ± SD from three independent experiments (n = 3). *P < 0.05, **P < 0.01, *P < 0.001 (one-way ANOVA with Bonferroni’s *post hoc* test).

#### 3.4.2 CPD inhibits the NOX2/ROS/PAD4 signaling axis

PMA stimulation triggered a robust ∼8.5-fold increase in intracellular ROS levels over the control (P < 0.001), quantitatively confirmed by flow cytometry (Figure 6C), and a burst that was visually apparent (Figure 6D). CPD pre-treatment significantly and dose-dependently suppressed this ROS burst. Furthermore, genetic knockdown of NOX2 (Si-NOX2) similarly potently attenuated ROS generation (2.9 ± 0.25, P < 0.001), confirming NOX2 as a principal source of ROS. At the molecular level, Western blot analysis revealed that PMA induction significantly upregulated the protein expression of NOX2, PAD4, and its product CitH3. CPD pre-treatment resulted in a dose-dependent downregulation of this entire signaling module. Crucially, Si-NOX2 transfection recapitulated the effects of CPD, markedly suppressing the expression of PAD4 and CitH3 (Figures 6E,F).

## 4 Discussion

Data from this randomized clinical trial, together with histological and ultrastructural observations of human perianal granulation tissue, collectively reveal that topical ultrasonically nebulized CPD significantly shortens anal fistulotomy wound healing and relieves local inflammatory manifestations. Reduced NET deposition visualized by TEM and immunofluorescence removes a core driver of sustained tissue inflammation, breaking the self-perpetuating inflammatory loop that stalls postoperative tissue repair.

Consistent with the NET-suppressive phenotype observed in patient granulation tissue, primary human neutrophil *in vitro* assays further clarify the underlying NOX2/ROS/PAD4 signalling cascade. CPD concentration-dependently blunts PMA-triggered ROS burst, a well-recognized upstream inducer of NF-κB transcriptional activation (Geng et al., 2024; Liu et al., 2024; Xue et al., 2025). As NF-κB acts as a master transcription factor controlling PAD4 expression (Dragoni et al., 2025; Li R. et al., 2024), suppressed NF-κB phosphorylation further lowers cellular PAD4 protein abundance and subsequent histone citrullination, ultimately limiting NET extrusion. This mechanistic chain is supported by published evidence of CPD’s key metabolites: berberine and tanshinone IIA both exert NF-κB inhibitory effects to restrict neutrophil inflammatory activation (Li J. et al., 2024; Wang et al., 2024; Gong et al., 2020; Mao et al., 2025).

This multi-layer regulatory profile explains the unique therapeutic merit of the multi-metabolite botanical drug, distinct from single-target synthetic agents. In surgical wound microenvironments, inflammation and NET formation form a self-amplifying pathological cycle (James et al., 2024; Michael et al., 2025); CPD interrupts this cascade at multiple regulatory nodes to establish a permissive microenvironment for tissue regeneration.

The increased CD31-positive capillary density provides a mechanistic explanation for accelerated tissue regeneration in the CPD group. The suppression of IL-6—a known VEGF antagonist (Arribas-Lopez et al., 2022; Choi et al., 2023)—coupled with the direct protection of the endothelium from NET-mediated damage (Lu et al., 2025), may synergistically promote vascular regeneration, a process indispensable for successful healing.

Beyond validating the clinical value of this multi-component botanical drug, our clinical and cellular data further support NETosis suppression as a viable therapeutic strategy for refractory surgical wounds, consistent with prior preclinical observations focused on chronic skin injury (Huang et al., 2025). The multi-modal regulatory characteristic of CPD offers translational advantage over single-target agents, and the core mechanistic logic may extend to other NETosis-associated conditions, such as diabetic ulcers and autoimmune disorders.

Nevertheless, this study has several inherent limitations. First, all mechanistic data are derived from human granulation tissue and primary neutrophil *in vitro* models, lacking supporting animal *in vivo* validation, which we plan to address in follow-up NOX2/ROS/PAD4 pathway verification experiments. Second, NET identification solely relied on CitH3 and CD66 staining; MPO or NE co-staining would improve detection specificity in future work. Third, the maximal non-cytotoxic concentration of 200 μg/mL used in cell culture exceeds the predicted local tissue drug level after topical nebulization, and subsequent low-concentration gradients plus tissue drug quantification will confirm physiological relevance. Finally, this single-center trial only assessed short-term wound outcomes, with no long-term follow-up for anal fistula recurrence and scar formation. Multi-center cohorts will be established to validate our clinical observations in further research.

## 5 Conclusion

In summary, our integrated design combining a randomized clinical trial with mechanistic assays demonstrates that CPD accelerates post-fistulotomy wound repair by ameliorating the local inflammatory and attenuating NETosis via modulation of the neutrophil ROS/NF-κB/PAD4 signalling cascade. These observations provide a pharmacological basis for the clinical application of CPD and identify NETosis suppression as a promising strategy for refractory surgical wounds.

## Data Availability

The original contributions presented in the article/Supplementary Material. Individual-level raw clinical data cannot be publicly released due to patient-privacy and ethical restrictions. De-identified aggregated datasets supporting the conclusions are available from the corresponding authors upon reasonable written request, subject to institutional ethics review.
