# Supplementary material for: Compound phellodendron decoction promotes post-fistulotomy wound healing by downregulating neutrophil extracellular traps formation: evidence from a randomized trial and mechanistic studies
Source: Front Pharmacol. 2026 Sep 15;17:1732119. doi: 10.3389/fphar.2026.1732119 (PMC13619825; doi:10.3389/fphar.2026.1732119)
Supplement: Supplementary file 1 [file DataSheet1.docx]

**Fingerprint Analysis of a Traditional Chinese Medicine Formula by UPLC-MS/MS——Project Report**

**1. Experimental Materials**

1. **Reagents and Materials**
   Chromatographic grade acetonitrile and methanol were purchased from Merck Group (Darmstadt, Germany). Chromatographic grade formic acid (purity 96%) was purchased from Tedia Company (Ohio, USA). Analytical grade methanol and 95% ethanol were purchased from China National Medicines Group Reagent Co., Ltd. (Shanghai, China). Ultrapure water was prepared using a Milli-Q water purification system (Millipore, Bedford, MA, USA). Reference standards Calceolarioside B (Batch No.: 18862), Matrine (Batch No.: 13509), Tanshinone IIA (Batch No.: 13163), Ginsenoside Rg1 (Batch No.: 13819), and Ferulic acid (Batch No.: 18905-G240701) were all purchased from Shanghai Standard Technology Service Co., Ltd. Berberine (Batch No.: O20IB229498) was purchased from Shanghai Yuanye Bio-Technology Co., Ltd.
2. **Herbal Materials**
   30g Phellodendron chinense cortex, 25g Sophora flavescens root, 15g Salvia miltiorrhiza root, 10g Panax notoginseng root, and 20g Angelica sinensis root were purchased from the Bozhou Chinese Herbal Medicine Wholesale Market.

**1.3 Instruments and Equipment**
Waters ACQUITY Ultra-Performance LC-Synapt G2 quadrupole time-of-flight (Q/TOF) liquid chromatography-mass spectrometry system, Waters Corporation (Milford, USA). Equipped with an ESI ion source and a Q-TOF quadrupole time-of-flight mass spectrometer. Instrument control and LC-MS data acquisition were performed using MassLynx 4.1 data analysis software.

KQ-250DB numerical control ultrasonic cleaner (Kunshan Ultrasonic Instrument Co., Ltd.); Sartorius BP211D electronic balance (Sartorius, Germany); Avanti J-25I centrifuge (Beckman, USA); VDRTEX-5 vortex mixer (Jiangsu, China); Eppendorf micro pipettes (Eppendorf, Germany).

**2. Experimental Methods**

**2.1 Preparation of Test Solution**
The formula: 30g Phellodendron chinense cortex, 25g Sophora flavescens root, 15g Salvia miltiorrhiza root, 10g Panax notoginseng root, 20g Angelica sinensis root, totaling 100g of sample, was added to an appropriate amount of ultrapure water and extracted by conventional heat reflux for 20 min. This process was repeated twice, and the extracts were combined. Then, 1 mL of the extract was transferred to a 1.5 mL centrifuge tube (this was done in parallel for 6 replicates), centrifuged at 4000 rpm/min for 5 min, and filtered through a 0.22 μm membrane to obtain 6 test solutions.

**2.2 Preparation of Reference Standard Solutions**
Accurately weighed amounts of Calceolarioside B (6.62 mg), Matrine (6.06 mg), Tanshinone IIA (5.28 mg), Ginsenoside Rg1 (7.91 mg), Ferulic acid (9.20 mg), and Berberine (6.14 mg) were placed separately into 5 mL volumetric flasks, made up to volume with methanol, and mixed well to obtain stock solutions. These were then diluted serially to establish calibration curves.

**2.3 Instrumental Analysis Conditions**
**Chromatographic conditions:** Column: ACQUITY UPLC™ HSS C18 column (100 × 2.1 mm i.d., 1.8 μm; Waters Co., Milford, MA, USA), equipped with an Acquity BEH C18 VanGuard pre-column (1.7 μm, 5 mm × 2.1 mm); Column temperature: 45 °C; Flow rate: 0.4 mL/min; Injection volume: 1 μL; Mobile phase: 0.1% formic acid in water (A) and acetonitrile (B), with gradient elution as follows: 0–5 min, 5%–20% B; 5–8 min, 20%–30% B; 8–11 min, 30%–60% B; 11–20 min, 60%–95% B.

**Mass spectrometric detection** was performed using an Acquity Synapt G2 QTOF tandem mass spectrometer (Waters, UK) interfaced with the UPLC system via an ESI source and controlled by MassLynx software version 4.1 (Waters, UK). The ESI source was operated in both positive (ESI+) and negative (ESI-) ionization modes. The optimized conditions for maximum metabolite response were as follows: capillary voltage, -2.5 kV (ESI-) or +3 kV (ESI+); sampling cone, -30 V (ESI-) or +30 V (ESI+); extraction cone, -4.0 V (ESI-) or +5.0 V (ESI+); source temperature, 120°C; desolvation temperature, 450°C; cone gas (nitrogen) flow, 50 L/h; desolvation gas (nitrogen) flow, 800 L/h. Collision gas was argon. Leucine-enkephalin (2 ng/mL) was used as the lock mass, generating reference ions at m/z 554.2615 (ESI-) or m/z 556.2771 (ESI+) via a 5 μL/min lock spray, to ensure mass accuracy during analysis. Data were acquired in centroid mode. The MSE method used two scan functions. In function 1, the parameters were set as: m/z 50-1200; scan duration 0.25 sec; interscan delay 0.02 sec; collision energy ramp 4 V. In function 2, the parameters were: scan range: m/z 100-1200; scan duration 0.25 sec; interscan delay 0.02 sec; collision energy 10-30 V. Data acquisition and processing were performed using Waters MassLynx version 4.1.

**3. Experimental Results**

**3.1 Linearity Results**
Using concentration as the horizontal coordinate and peak area as the vertical coordinate, linear regression was performed. The results are shown in Table 1 and Figures 1-6, indicating good linear relationships for the reference standard solutions within the linear ranges.

Table 1-1-2 Linear regression equation of three index components

| 成分 | Linearrange(mg.mL^-1^) | Regressionequation | R^2^ | Rt(min) |
| --- | --- | --- | --- | --- |
| Matrine (+) | 0.0024-0.48 | y = 165200x + 2302.8 | 0.9967 | 1.92 |
| Ferulic acid (-) | 0.0184-0.736 | y = 2070.8x + 28.045 | 0.9995 | 6.43 |
| Calceolarioside B (-) | 0.00264-0.528 | y = 36164x + 258.71 | 0.9997 | 6.71 |
| Ginsenoside Rg1 (-) | 0.00316-0.632 | y = 7460.9x + 105.4 | 0.9990 | 8.25 |
| Berberine (+) | 0.00244-0.488 | y = 2E+06x + 34617 | 0.9991 | 9.01 |
| Tanshinone IIA (+) | 0.00208-0.408 | y = 70611x + 116.34 | 0.9994 | 15.5 |

Standard curve for Matrine

|  |  |
| --- | --- |
| 0.0024 | 1134.75 |
| 0.012 | 5029 |
| 0.024 | 7052.95 |
| 0.12 | 22398 |
| 0.24 | 41715.2 |

Fig1 Standard curve for Matrine

Standard curve for Ferulic acid

|  |  |
| --- | --- |
| 0.0184 | 48.5 |
| 0.0368 | 105.8 |
| 0.184 | 429.5 |
| 0.368 | 790.933 |
| 0.736 | 1547 |

**Figure** 2 Standard curve for Ferulic acid

Standard curve for Calceolarioside B

|  |  |
| --- | --- |
| 0.00264 | 123.2 |
| 0.0132 | 742.8 |
| 0.0264 | 1337.2 |
| 0.132 | 5179.9 |
| 0.264 | 9795.9 |
| 0.528 | 19316 |

**Figure 3** Standard curve for Calceolarioside B

Standard curve for Ginsenoside Rg1

|  |  |
| --- | --- |
| 0.00316 | 35.0667 |
| 0.0158 | 244.9 |
| 0.0316 | 404.7 |
| 0.158 | 1325.35 |
| 0.316 | 2425.35 |
| 0.632 | 4826 |

**Figure 4** Standard curve for Ginsenoside Rg1

Standard curve for Berberine

|  |  |
| --- | --- |
| 0.00244 | 19623.6 |
| 0.0122 | 58226.1 |
| 0.0244 | 94209.3 |
| 0.122 | 280991 |
| 0.244 | 515072 |
| 0.488 | 981954 |

**Figure 5** Standard curve for Berberine

Standard curve for Tanshinone IIA

|  |  |
| --- | --- |
| 0.00208 | 167 |
| 0.0104 | 833.15 |
| 0.0208 | 1858.3 |
| 0.104 | 7050.1 |
| 0.208 | 15127.5 |
| 0.408 | 28852 |

**Figure 6** Standard curve for Tanshinone IIA

**3.2 Determination of Sample Content**

Table 1-1-7 The results of sample content experiment（mg/g）

| No. | Component | Content (mg/g) |
| --- | --- | --- |
| 1 | Matrine | 21.3 ± 2.43 |
| 2 | Ferulic acid | 0.67 ± 0.071 |
| 3 | Calceolarioside B | 7.3 ± 0.64 |
| 4 | Ginsenoside Rg1 | 79.6 ± 7.43 |
| 5 | Berberine | 4.4 ± 0.61 |
| 6 | Tanshinone IIA | 19.9±4.87 |

**3.3 Specificity Investigation**

**As shown in Figures 7 and 8, the Base Peak Chromatograms (BPC) in positive and negative ion modes respectively, indicate minimal interference from extraneous peaks, demonstrating good specificity for the six index components under this detection method**

Figure 7 Base peak ion chromatograms (BPC) of sample obtained by UPLC-MS/MS in the negative ion mode. A: Mixed standard; B: Sample; C: Methanol blank control. 1: Ferulic acid; 2: Calceolarioside B; 3: Ginsenoside Rg1.

Figure 8 BPC of sample obtained by UPLC-MS/MS in the positive ion mode. A: Mixed standard; B: Sample; C: Methanol blank control. 1: Matrine; 2: Berberine; 3: Tanshinone IIA.

**3.4 Sample Fingerprint**

**As shown in Figures 9 and 10, the fingerprint chromatograms of the 6 parallel-prepared test solutions in negative and positive ion modes, respectively, are characteristic and identifiable.**

Figure 9 BPC of 6 samples obtained by UPLC-MS/MS in the negative ion mode

Figure 10 BPC of 6 samples obtained by UPLC-MS/MS/MS in the positive ion mode

**3.5 MS/MS Spectra of the Six Index Components**

**Figure 11** MS/MS spectrum of Matrine

**Figure 12** MS/MS spectrum of Ferulic acid

**Figure 13** MS/MS spectrum of Calceolarioside B

**Figure 14** MS/MS spectrum of Ginsenoside Rg1

**Figure 15** MS/MS spectrum of Berberine

**Figure 16** MS/MS spectrum of Tanshinone IIA
